# Proposed Law for the Extermination of Cattle-tuberculosis in Denmark

**Published:** 1898-08

**Authors:** 


PROPOSED LAW FOR THE EXTERMINATION OF CATTLE-TUBERCULOSIS IN DENMARK. The draft includes, according to the *Milch-Zeitung*, two principal parts: (1) in regard to the importation of cattle from abroad, and (2) in regard to domestic regulations. Further, the annual sum of 100,000 kr. shall be placed at the disposition of the Minister of Agriculture in order that he may distribute aid in the form of tuberculin-inoculation to cattle-owners, breeding and agricultural associations. Cattle from abroad can be imported only at certain places designated by the Minister of Agriculture. They must be brought immediately to quarantine stations, and, at the latest, three days after arrival be subjected to a tuberculin-inoculation. Those animals, however, which are to be slaughtered within ten days after arrival are not subjected to inoculation. The cost of maintaining quarantine stations and of tuberculin-inoculations is to be borne by the government, because the tests are made in the interests of the general public.

The domestic regulations propose that cows with tuberculosis of the udder be slaughtered by public enactment; further, that all milk-dealers and dairies shall sell only milk and buttermilk which has been warmed to 85° C.; the same regulation to apply to old milk and buttermilk imported from abroad. In regard to Pasteurizing the buttermilk no provision seems to be made provided the cream is Pasteurized.—*Idem.*

